# Spatiotemporal Characterization of Anterior Segment Mesenchyme Heterogeneity During Zebrafish Ocular Anterior Segment Development

**DOI:** 10.3389/fcell.2020.00379

**Published:** 2020-05-27

**Authors:** Kristyn L. Van Der Meulen, Oliver Vöcking, Megan L. Weaver, Nishita N. Meshram, Jakub K. Famulski

**Affiliations:** Department of Biology, University of Kentucky, Lexington, KY, United States

**Keywords:** periocular mesenchyme, anterior segment, anterior segment dysgenesis, neural crest, *pitx2*, *foxc1*

## Abstract

Assembly of the ocular anterior segment (AS) is a critical event during development of the vertebrate visual system. Failure in this process leads to anterior segment dysgenesis (ASD), which is characterized by congenital blindness and predisposition to glaucoma. The anterior segment is largely formed via a neural crest-derived population, the Periocular Mesenchyme (POM). In this study, we aimed to characterize POM behaviors and transcriptional identities during early establishment of the zebrafish AS. Two-color fluorescent *in situ* hybridization suggested that early AS associated POM comprise of a heterogenous population. *In vivo* and time-course imaging analysis of POM distribution and migratory dynamics analyzed using transgenic zebrafish embryos (Tg[*foxc1b:*GFP], Tg[*foxd3:*GFP], Tg[*pitx2:*GFP], Tg[*lmx1b.1:*GFP], and Tg[*sox10:*GFP]) revealed unique AS distribution and migratory behavior among the reporter lines. Based on fixed timepoint and real-time analysis of POM cell behavior a comprehensive model for colonization of the zebrafish AS was assembled. Furthermore, we generated single cell transcriptomic profiles (scRNA) from our POM reporter lines and characterized unique subpopulation expression patterns. Based on scRNA clustering analysis we observed cluster overlap between neural crest associated (*sox10*/*foxd3*), POM (*pitx2*) and finally AS specified cells (*lmx1b*, and *foxc1b*). scRNA clustering also revealed several novel markers potentially associated with AS development and/or function including *lum*, *fmoda*, *adcyap1b*, *tgfbi*, and *hmng2*. Taken together, our data indicates that AS-associated POM, or Anterior Segment Mesenchyme (ASM), is not homogeneous but rather comprised of several subpopulations with differing colonization patterns, migration behavior, and transcriptomic profiles.

## Introduction

Vertebrate cranial development has benefitted significantly from the evolutionary addition of the multipotent neural crest cells (NCC). Originating in the dorsal neural ectoderm of the folding neural tube, neural crest cells (NCC) undergo an epithelial-to-mesenchymal transition, detaching themselves from the epithelial sheet and migrating in distinct streams to invade regions all over the developing embryo. NCCs ultimately go on to form diverse mesodermal derivatives including cartilage, myofibroblasts, neurons, and glial cells (Trainor and Tam, 1995; Langenberg et al., 2008; Williams and Bohnsack, 2015). In the developing cranial region, migrating NCCs come together with lateral plate mesoderm to surround the developing optic cup and form the Periocular Mesenchyme (POM) (Trainor and Tam, 1995; Langenberg et al., 2008; Williams and Bohnsack, 2015). POM subsequently contribute to the development of the ocular anterior segment (AS) (Supplementary Figure S1) (Fuhrmann et al., 2000; Creuzet et al., 2005; Williams and Bohnsack, 2015; Akula et al., 2018). The AS, comprising of the cornea, lens, iris, ciliary body, and drainage structures of the iridocorneal angle, is essential for the function of the visual system. The AS focuses light onto the retina while maintaining intraocular homeostasis.

Anterior segment development begins after the establishment of the optic cup, when POM cells migrate into the periocular space between the retina and the newly established corneal epithelium (Creuzet et al., 2005; Cavodeassi, 2018). These mesenchymal cells will eventually differentiate into the corneal stroma and endothelium, iris and ciliary body stroma, and the iridocorneal angle, amongst others. Mis-regulation of POM migration or function has been associated with congenital blinding disorders under the term anterior segment dysgenesis (ASD). ASD includes, alone or in combination, corneal opacity, iris hypoplasia, polycoria, corectopia, posterior embryotoxon, juvenile glaucoma, and disorders including Peter’s Anomaly and Axenfeld-Rieger Syndrome (Gould et al., 2004; Volkmann et al., 2011; Akula et al., 2018). These rare autosomal dominant disorders, in addition to ASD phenotypes, also often exhibit systemic issues including dental malformations and craniofacial defects (Volkmann et al., 2011; Bohnsack et al., 2012; Ji et al., 2016). In addition to congenital diagnoses, failure of proper AS formation may also result in a predisposition to ASD later in life. Despite its fundamental role in the establishment of the AS, little is understood about the mechanisms governing POM specification, migration or differentiation.

The most common mutations seen in ASD patients involve the transcription factor *pitx2* (Paired-like homeodomain) (Ji et al., 2016), as well as *foxc1* (Forkhead Box c1) (Berry et al., 2006; Bohnsack et al., 2012; Reis et al., 2012; Chen and Gage, 2016; Seo et al., 2017). Loss of function of either *pitx2* or *foxc1* has been shown to result in ASD phenotypes in mice and zebrafish (Berry et al., 2006; Liu and Semina, 2012; Reis et al., 2012; Chen and Gage, 2016; Ji et al., 2016; Seo et al., 2017; Hendee et al., 2018). *Pitx2* in particular has been associated with the survival and migration of NCCs, as well as the development of the optic stalk, establishment of angiogenic privilege within the cornea, and craniofacial development (Evans and Gage, 2005; Bohnsack et al., 2012; Liu and Semina, 2012; Gage et al., 2014; Chawla et al., 2016; Chen and Gage, 2016; Ji et al., 2016; Hendee et al., 2018). *Foxc1* and *pitx2* are also known to interact with one another, and their expression is regulated by retinoic acid signaling (Matt et al., 2005; Chawla et al., 2018). Not surprisingly, mutations in NCC regulatory genes have also been associated with ASD. *Foxd3* (Forkhead Box d3) has been implicated in ASD (Volkmann Kloss et al., 2012) and is known to regulate early NCC specification, migration and long-term cell survival (Lister et al., 2006; Stewart et al., 2006; Drerup et al., 2009; Wang et al., 2011). *Sox10* (SRY-Box 10), another key regulator of the NCC population (Dutton et al., 2001; Creuzet et al., 2005; Langenberg et al., 2008; Drerup et al., 2009; Williams and Bohnsack, 2015), is critical for NCC migration and viability during early development (Dutton et al., 2001). Finally, *lmx1b* (LIM homeobox Transcription Factor 1 beta) is associated with Nail-Patella syndrome and glaucoma predisposition (McMahon et al., 2009; Liu and Johnson, 2010). *Lmx1b* is expressed within the developing cornea, iris, ciliary bodies, and trabecular meshwork of the iridocorneal angle in mice (McMahon et al., 2009; Liu and Johnson, 2010) and is essential for POM migration in zebrafish (McMahon et al., 2009). While several genes have been linked to POM or ASD, few studies to date have shed light on how POM cells, migrate to and participate in AS formation.

One signaling molecule that is known to be involved in cranial neural crest cell migration and anterior segment specification is Retinoic Acid (RA). RA, a metabolite of vitamin A, is instrumental for the overall development of the eye. RALDHs (RA-synthesizing enzyme retinaldehyde dehydrogenases) are expressed in a gradient through the eye, specifically in the retina, cornea, RPE, and lens (Matt et al., 2005, 2008; Lupo et al., 2011). RA produced in these areas diffuses out toward the anterior space of the developing eye, which will be populated by the POM. RA signaling is activated through the heterodimer receptors RARα/RARβ and RARα/RARγ expressed within the POM (Matt et al., 2005, 2008; Lupo et al., 2011). This signal is a vital determinate in eye morphogenesis. Activation of these receptors helps to control *eya2* dependent apoptosis in the POM as well as control the expression levels of *foxc1* and *pitx2* and therefore anterior segment development (Matt et al., 2005, 2008). NCC-specific deactivation of RARα, RARβ, and RARγ results in a complete loss of *pitx2* in the POM and AS, while increased RA signaling leads to an increase in *pitx2*, *foxc1a*, and *lmx1b.1* (Matt et al., 2008; Lupo et al., 2011). Though not explicitly explored in this study, RA signaling is a crucial component of cell migration and overall eye morphogenesis.

Although information about the anatomy of the anterior segment in vertebrates and anatomical consequences of POM regulatory gene mutations has been well documented, few studies have investigated the mechanism of development for AS structures overall. Specifically, little is known about when or how POM cells acquire their AS targeting, behave during migration, interact with one another, and finally, specify into various AS structures. Within this study, we aimed to characterize the developmental underpinnings that drive the formation of the AS. Using zebrafish embryos, we characterized the precise migration patterns and transcriptional profiles of AS associated POM cells (ASM). We specifically examined ASM gene expression as well as cellular distribution by taking advantage of POM-associated transgenic lines; Tg[*foxc1b:*GFP], Tg[*foxd3:*GFP], Tg[*pitx2*:GFP], Tg[*lmx1b.1:*GFP], and Tg[*sox10:*GFP]. In doing so, we have cataloged distribution, migratory dynamics, and population size of ASM during early AS development. Furthermore, single cell transcriptomic comparison of isolated ASM cells revealed four specific clusters, each associated with potentially novel AS regulatory genes. Our findings indicate that AS-associated POM is composed of several subpopulations, each identifiable by their own distributions, migratory patterns, and gene expression profile.

## Results

### POM-Associated Genes Exhibit Unique Expression Patterns During Early Establishment of the Anterior Segment

**FIGURE 1:**
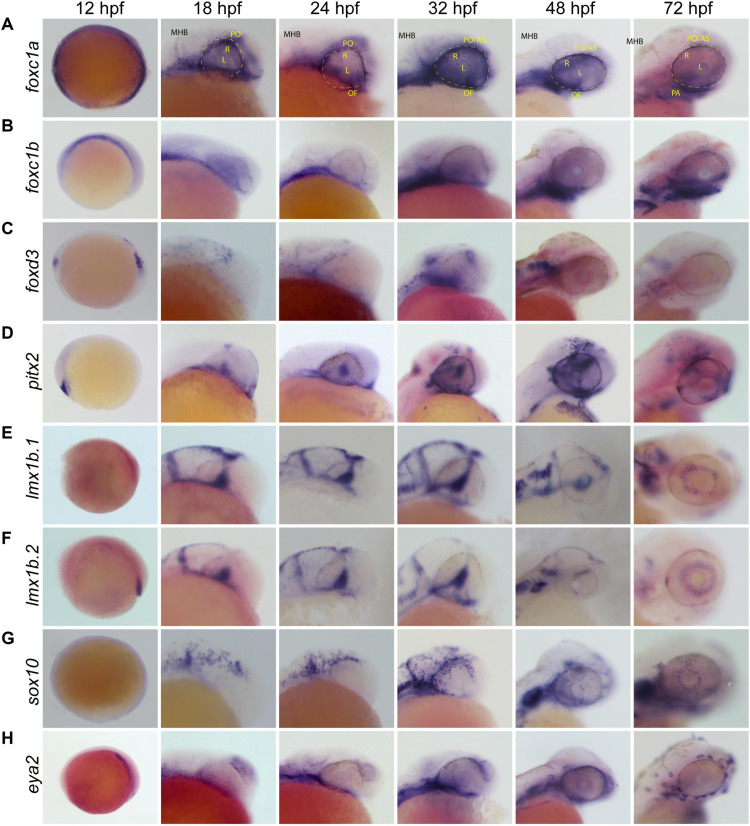
Whole Mount *In Situ* Hybridization (WISH) of known POM and neural crest-related marker genes. Whole-mount *in situ* hybridization for POM marker gene mRNA expression patterns were observed during early to late stage eye development in the lateral view. POM genes examined were **(A)** *foxc1a*, **(B)** *foxc1b*, **(C)** *foxd3*, **(D)** *pitx2*, **(E)** *lmx1b.1*, **(F)** *lmx1b.2*, **(G)** *sox10*, and **(H)** *eya2. Foxc1a*, *foxc1b*, *pitx2*, *eya2*, and *sox10* in particular show strong expression surrounding the optic cup and on the surface of the anterior segment from 24 to 72 hpf. *Lmx1b.1* and *lmx1b.2* expression is detected within the anterior segment, surrounding the lens by 48–72 hpf. MHB, midbrain-hindbrain boundary; R, retina; L, lens; OV, optic vesicle; PO, periocular space (outlined with dashed line); OF, optic fissure; AS, anterior segment.

With several genes being implicated in regulating POM migration and identity, we first chose to carefully characterize patterns of their expression during zebrafish ocular morphogenesis (12–72 hpf). Whole Mount *In Situ* Hybridization (WISH) using embryos aged 12, 18, 24, 32, 48, and 72 hpf revealed that POM-related genes *foxc1a*, *foxc1b*, *eya2*, *foxd3*, *pitx2*, *sox10*, *lmx1b.1* and *2*, display both overlapping and individualized expression patterns within their originating neural crest streams and surrounding the AS (Figure 1). Several POM-related genes showed expression at 12 hpf, the earliest time point we assayed, suggesting that POM acquire their identity early, perhaps immediately following their delamination from the neural tube. As the optic cup begins to take shape (18 hpf), *foxc1a*, *foxc1b*, and *sox10* expressing POM cells are already visible within the craniofacial space. At the same time, *pitx2* expression is absent from periocular regions and presents primarily in the lens. By 24 hpf we observed various degrees of periocular expression of all the aforementioned POM-associated genes. *Foxc1a* displays the prototypical POM expression pattern with signal extending from the forebrain and into the surrounding periphery, and on top of, the retina by 32 hpf (Figure 1A and Supplementary Figure S1). Similar, albeit much weaker expression of *foxc1b* and *eya2* can be observed at 24 and 32 hpf (Figures 1B,H). Both *foxd3* and *sox10* display partial periocular patterns of expression, predominantly in the temporal regions at 24 hpf and more homogenously by 32 hpf (Figures 1C,G). *Pitx2* also displays strong periocular expression staining at 32 hpf but is also uniquely expressed in the lens (Figure 1D). Periocular-like expression patterns become clearest by 48 hpf for *foxc1a*, *foxc1b*, *eya2*, *pitx2*, and *sox10*. *Foxd3* periocular expression is significantly diminished by 48 hpf. By 72 hpf only *foxc1a*, *pitx2*, *sox10*, and *eya2* still exhibit strong periocular expression. In spite of the implicated role of *lmx1b* genes in pathogenic features of the Nail-Patella Syndrome (McMahon et al., 2009; Liu and Johnson, 2010), both *lmx1b.1* and *lmx1b.2* genes did not display classical AS expression patterns in our WISH assay at early timepoints, 12–48 hpf, but are detected in the AS at later stages, 72 hpf+ (Figures 1E,F). Taken together, we observe that POM-associated genes are not uniformly co-expressed in POM cells during early AS formation. Early POM, 12–24 hpf, express high levels of *foxc1a*, and only at later stages of AS colonization, 24–32 hpf, do they initiate high levels of expression for *foxc1b*, *eya2*, and *pitx2*. POM cells which express *sox10/foxd3* display periocular patterns only after 24 hpf, suggesting they may arrive as a second wave. At late stages of AS colonization, 48–72 hpf, we observe the persistence of strong *foxc1a* expression and an upregulation of *foxc1b*, *pitx2*, and *eya2*. Between 48 and 72 hpf, *sox10* expression is detected throughout the AS but does not appear to increase significantly while *foxd3* expression is no longer detected in the AS after 48 hpf. Based on our WISH observations, we therefore hypothesized that AS colonizing POM populations are likely heterogenous.

### Co-expression Analysis Confirms Anterior Segment Mesenchyme Heterogeneity

**FIGURE 2:**
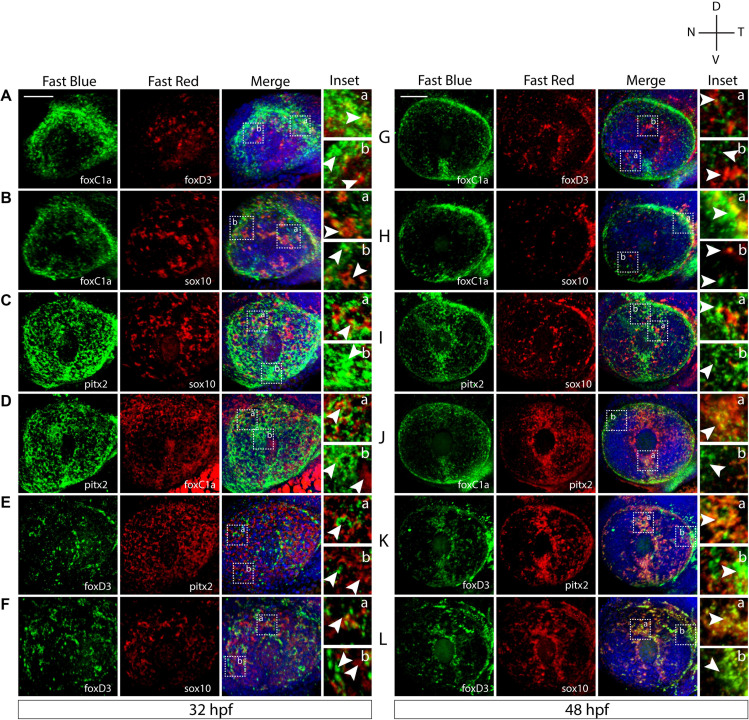
Two-color fluorescent *in situ* hybridization supports ASM heterogeneity. Two-color fluorescent WISH (FWISH) performed for all possible combinations of *foxc1a*, *foxd3*, *pitx2*, and *sox10* at 32 **(A–F)** and 48 hpf **(G–L).** DAPI is in blue. Lateral images of 3D reconstructions are displayed. White arrows within inset panels (dashed squares) display instances of individual **(b)** and co-expression **(a)**. Scale bar = 50 μm.

Based on our WISH study, we next sought to determine the extent of co-expression between the POM associated genes. To study these relationships, we performed two-color fluorescent whole mount *in situ* hybridization (FWISH) at 32 and 48 hpf. These timepoints represent early and intermediate steps of POM AS colonization. We focused our attention on the expression of *foxc1*, *foxd3*, *sox10*, and *pitx2* as they represent the best studied AS-associated POM marker genes. 3D confocal imaging qualitatively indicated that all these POM markers clearly exhibit both overlapping and individualized expression patterns at 32 hpf (Figures 2A–F). All of our described results are based on reproducible patterns observed in 12 hpf+ embryos from two independent experiments. At 32 hpf, *foxc1* expression appears restricted largely to the periphery of the AS while *sox10*, *foxd3*, and *pitx2* display varying degrees of expression throughout the dorsal, ventral, nasal, and temporal quadrants. *Pitx2* exhibited broad expression throughout the AS and a high degree of co-expression with *foxc1*, primarily in the dorsal quadrant, and *sox10*, throughout the entire AS, at 32 hpf (Figures 2C,D). *Foxc1* exhibited a high degree of co-expression with *pitx2* in the dorsal and ventro-temporal AS and partial co-expression with *foxd3* and *sox10* (Figures 2A,B,D). The expression of *sox10* was most pronounced in the dorsal AS and had the highest degree of co-expression with *foxd3* (Figures 2B,C,F). *Foxd3* expression was detected throughout the AS, albeit in fewer cells than the other markers, and exhibited a co-expression primarily with *sox10* and *pitx2* throughout the entire AS (Figures 2A,E,F). By 48 hpf, noticeably pronounced individualized expression patterns emerged (Figures 2G–L). *Pitx2* expression became restricted to the dorsal and ventral quadrants of the AS, while *foxc1* expression expanded into the entire AS (Figures 2G–K). Co-expression was still evident between *pitx2* and *foxc1*, but primarily in the dorsal and ventral quadrants. *Foxd3* and *sox10* maintained the same spatial pattern of expression as observed at 32 hpf and continued to have a high degree of co-expression, particularly surrounding the lens (Figure 2L). *Foxd3* and *sox10* continued to have only minor co-expression with *pitx2* or *foxc1* throughout the AS. We also analyzed expression of *eya2* and observed a high degree of co-expression with *pitx2* throughout the entire AS, and slight co-expression with all the other markers (Supplementary Figure S2). Taken together, we show that already at 32 hpf AS-associated POM do not exhibit a homogenous expression pattern of POM-associated regulatory genes. This suggest that the AS is colonized as an already heterogenous population rather than undergoing later diversification from a common progenitor.

### POM Cells Display Distinct Targeting Patterns During Early AS Colonization

**FIGURE 3:**
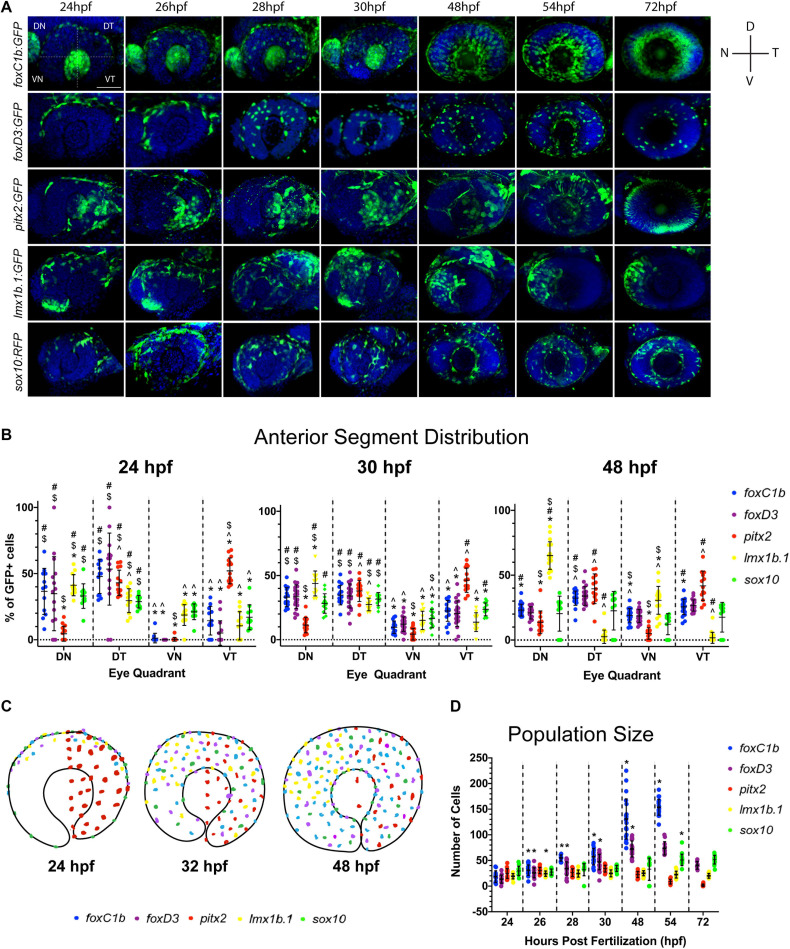
Periocular mesenchyme subpopulation distribution analysis. **(A)** 3D rendering of confocal stacks encompassing the AS in POM transgenic lines between 24 and 72 hpf. GFP+ cells are green, DNA was stained with DAPI (blue). DN, dorsal-nasal; DT, dorsal-temporal; VN, ventral-nasal; VT, ventral-temporal. Scale bar = 50 μm. **(B)** Distribution of quantified GFP+ cells within each AS quadrant (DN, DT, VN, and VT) at 24, 30, and 48 hpf. Distribution is represented as an average percentage of the total number of GFP+ in each quadrant of the AS for each transgenic line. Statistically significant difference (*p* < 0.05): * vs. DT, # vs. VN, $ vs. VT and ^ vs. DN. **(C)** Model of POM colonization at 24, 30, and 48 hpf. **(D)** Average AS cell population size for each transgenic line at 24–72 hpf. * indicates statistically significant (*p* < 0.05) change from previous time point.

Based on our FWISH results we hypothesized that the heterogenous population of AS progenitors may display unique migration patterns and behavior. Furthermore, key to our understanding of AS formation will be the awareness of when and how the POM colonize. To begin characterizing this process, we first took advantage of available transgenic lines known to label POM: Tg[*foxc1b:*GFP], Tg[*pitx2:*GFP], Tg[*lmx1b.1:*GFP], or NCC: Tg[*foxd3:*GFP], Tg[s*ox10:*GFP] (Matt et al., 2005, 2008; McMahon et al., 2009; Volkmann et al., 2011). These reporter lines enable single cell distribution analysis while also delineating lineage specification. Due to persistence of GFP protein, these lines do not necessarily represent active expression of their reporter driven promoter but do mark the lineage of POM/NCCs that have, at some point, expressed the respective POM-associated gene. To analyze AS colonization, transgenic embryos were fixed at key AS developmental stages (24, 26, 28, 30, 48, 56, and 72 hpf) and immunohistochemistry (IHC) was used to detect GFP. 3D confocal images of the AS were collected for each transgenic line at each timepoint and were employed to subsequently quantify distribution of the cells within the AS (Figure 3A). In particular, 3D rendered images of the AS were subdivided into four quadrants (Figure 3A, top left panel) and cells found in each were quantified at each timepoint.

Our assay revealed that initial colonization of the AS begins at approximately 22 hpf (data not shown) with GFP+ cells of the *pitx2-*derived population occupying the temporal AS regions (Figure 3A). At 24 hpf *foxc1b*, *foxd3*, and *lmx1b.1* derived GFP+ cells have begun to enter the AS across the dorsal most periocular regions, while *sox10-*derived cells begin to occupy all four regions. By 28 hpf all of the reporter lines, with the exception of *pitx2*, exhibit GFP+ cells primarily in the dorsal half of the AS. Tg[*foxc1b*:GFP] signal is also observed in the developing lens region up to 32 hpf, but was not considered for our analysis. Similarly, up to 32 hpf Tg[*lmx1b.1*:GFP] non-AS signal is observed in the dorsal-nasal region but is not considered in our analysis because it does not contribute to the AS. *Foxc1b*, *foxd3*, and *sox10* derived cells continue to spread to the ventral regions with roughly equal distribution throughout the AS by 48 hpf. Conversely, *pitx2*-derived cells remain exclusively associated with the temporal half of the AS while *lmx1b.1*-derived cells gradually re-distribute to occupy the nasal half of the AS. Starting at 54 hpf and continuing to 72 hpf, Tg[*pitx2:*GFP] expression turns off in POM cells and initiates in what is likely photoreceptor progenitor cells. Of note, our Tg[*pitx2*:GFP] transgenic line did not exhibit GFP signal in all regions of AS that were found to be positive for *pitx2* mRNA using FWISH (Figures 2, 3A). We hypothesize this discrepancy arises from the fact that ASM progenitor cells induce *pitx2* expression upon arrival at the AS, while the GFP+ cells are a lineage mark of the early *pitx2*+ progenitors. The enhancer element driving the Tg[*pitx2*:GFP] line, C4, may be no longer responsive in ASM cells at the later time points and therefore explain the lack of GFP signal in all of the *pitx2* expressing cells.

All of our observations were subsequently validated by quantification of each GFP+ population (Figure 3B). At 24 hpf the majority of POM cells are located within the dorsal half of the AS, with the exception of *pitx2*-derived cells. By 30 hpf we note a significant reduction in the proportion of *foxc1b*, *foxd3*, and *lmx1b.1*-derived cells in the dorsal half combined with significant increase of these cells in the ventral half. At 48 hpf, we found equal distribution of *foxc1b*, *foxd3*, and *sox10* derived cells within all regions of the AS, while *lmx1b.1*-derived cells become predominantly associated with the nasal half of the AS.

Overall, we observe an ordered pattern of AS colonization, as summarized in Figure 3C. The majority of POM cells, *foxc1b*, *foxd3*, and *sox10:*GFP-derived cells, enter the AS along the dorsal retina and progress ventrally, while the *pitx2:*GFP sub-lineage enters the AS temporally and remains exclusively within the temporal AS. *lmx1b.1*:GFP cells enter the AS dorsally and initially migrate ventrally but by 48 hpf become restricted to the nasal regions of the AS.

### Cell Proliferation Does Not Drive ASM Population Growth During AS Colonization

A distinct fluctuation in the total number of GFP+ cells within each transgenic line examined throughout the time course was also noted. While *lmx1b.1*, *pitx2*, and *sox10*-derived populations maintained a relatively consistent total number of cells, f*oxc1b* and *foxd3*-derived populations increased in size over time (Figure 3D). This is particularly evident in the *foxc1b-*population which increased size so significantly by 72 hpf that it was no longer quantifiable by our assay. Having documented the increase in population size of some ASM subpopulations, we next sought to understand the mechanisms responsible for this change. Specifically, we wanted to investigate whether the increase in population size of the *foxc1b:GFP* and *foxd3:GFP* subpopulations was the result of a continued influx of migratory cells into the AS or proliferation of cells already in the AS. Transgenic embryos were fixed at 32 and 48 hpf as this window in development sees the largest increase in population size. Proliferating cells were identified using pH3 antibody staining. 3D images of the AS were collected using a confocal microscope. Cells positive for both the GFP and the pH3 signal were quantified (Supplementary Figure S3). Little to no pH3 signal at either 32 or 48 hpf in *foxd3*:GFP or *foxc1b*:GFP embryos, or any of our transgenic lines, indicates that ASM cells are not actively dividing while migrating within the AS. Based on these data, we suggest that the increase in *foxc1b*:GFP and *foxd3*:GFP cell populations is the sole result of rapid and continuous migration of cells to the AS between 32 and 48 hpf.

### ASM Subpopulations Exhibit Unique Migratory Behavior

**FIGURE 4:**
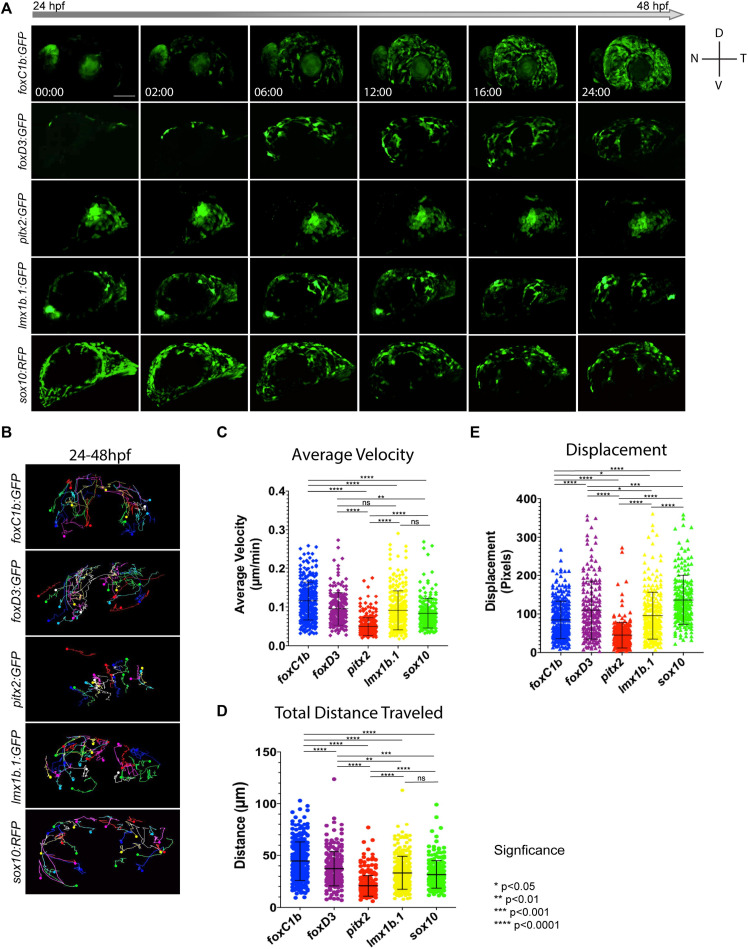
*In vivo* 4D imaging of POM anterior segment colonization. **(A)** 4D *in vivo* imaging of the AS conducted between 24–48 hpf using POM transgenic lines. Time stamp hours:minutes. Scale bar = 50 μm. **(B)** Individual cell tracking for each POM transgenic line reveals migratory patterns during early AS colonization. Solid spheres indicate terminal end of track. **(C–E)** Cell tracking measurements of average migratory velocity (ANOVA *p* < 0.0001), total migration distance (ANOVA *p* < 0.0001), and migratory displacement within the AS (ANOVA *p* < 0.0001).

In addition to cellular distribution over time, we also sought to catalog the migratory behavior of ASM cells. Specifically, we aimed to determine if they behaved in a similar fashion to cranial NCCs. Hence, we tracked their migration within the AS using *in vivo* 4D imaging. Using this approach we documented migration of *foxc1b:*GFP, *foxd3:*GFP, *pitx2:*GFP, *lmx1b.1:*GFP, and *sox10:*GFP-derived cells (Figure 4A). Qualitative examination of our data indicated that in all the transgenic lines, ASM cells migrated in a stochastic manner. This suggests that similar to cranial NCCs, ASM cells lack leader/follower cell identities or chain migration behavior (Figure 4A and Supplementary Movies S1–S5). The 4D data sets mirrored the cellular distribution trends we quantified in our previous time course assay (Figure 3). As expected based on our distribution studies, both *pitx2* and *lmx1b.1*:GFP cells displayed very specific distributions within the AS (Figure 4B), while *foxc1b*, *foxd3*, and *sox10:*GFP had more homogenous distribution patterns.

To analyze individual migratory behavior, we performed individual cell tracking. As expected, *foxc1b:*GFP, *foxd3:*GFP, *lmx1b.1:*GFP and *sox10*:GFP*-*derived cells migrated in a general dorsal to ventral pattern (Figure 4B and Supplementary Movies S6–S10). *Pitx2:*GFP cells migrated in a generally temporal to nasal direction (Figure 4B). Tracked cells were analyzed for their total distance traveled, directed migration, and velocity. *Foxc1b-*derived ASM had the highest velocities (0.115 ± 0.048 μm/min) while the cells of the *pitx2-*derived subpopulation were the slowest (0.049 ± 0.023 μm/min) (Figure 4C). *Foxd3*, *lmx1b.1*, and *sox10-*derived ASM cells had similar velocities (0.095 ± 0.042; 0.091 ± 0.050; and 0.079 ± 0.033 μm/min). When examining total distance traveled, *foxc1b-*derived cells displayed the farthest distances overall (44.667 ± 18.531 μm), while *pitx2-*derived cells exhibited the shortest (20.719 ± 9.936 μm) (Figure 4D). *Foxd3*, *lmx1b.1*, and *sox10*-derived ASM all exhibited similar overall distances traveled (37.339 ± 16.769; 33.267 ± 15.869; and 31.688 ± 13.289 μm). Differences in total migratory distance and velocity based on the AS quadrant of entry were also compared. Only *foxc1b-*dervied cells originating in the dorsal temporal quadrant showed differences in migration displaying shorter total distance traveled and slower velocity (data not shown). Based on analysis of migration velocity and distance traveled we conclude that ASM exhibit of a mixture of migration patterns and behaviors. This further supports our hypothesis that during colonization of the AS, the ASM are a heterogenous population.

Lastly, we measured the degree of directed migration by examining individual cell displacement within the AS (Figure 4E). Displacement was used as a measure of purposeful, or targeted, migration. We saw that cells within the *sox10* subpopulation showed the highest overall displacement (136.885 ± 63.327 pixels), followed by cells in the *foxd3* subpopulation (109.70 ± 75.434 pixels). Displacement of *foxc1b* and *lmx1b.1* was found to be 84.632 ± 49.223 and 95.559 ± 61.285 pixels, respectively. Similar to our previous observations for velocity and distance, the *pitx2* subpopulation exhibited the least amount of displacement (44.698 ± 33.008 pixels). Our data indicate that all ASM cells are highly migratory, but that as observed for POM marker gene expression, there is heterogeneity when comparing the various reporter lines.

### ASM Subpopulations Cluster According to Developmental Transcriptomic Profiles

Having observed the AS-associated POM subdivide into several ASM subpopulations, based on distribution and migratory behavior, our final goal was to analyze single cell transcriptomic profiles of ASM cells during AS colonization. To do so, we dissected the eyes off of our transgenic embryos at 48 hpf, isolated GFP+ cells via FACS, and subsequently employed the Chromium 10X genomics platform to generate single cell transcriptomes (scRNA). Resulting transcriptomes were sequenced using Illumina technology and analyzed using Cell Ranger3.1 and Loupe software. In total we sequenced 2,460 individual cells with per cell reads of greater than 100,000 (Supplementary Table S2).

**FIGURE 5:**
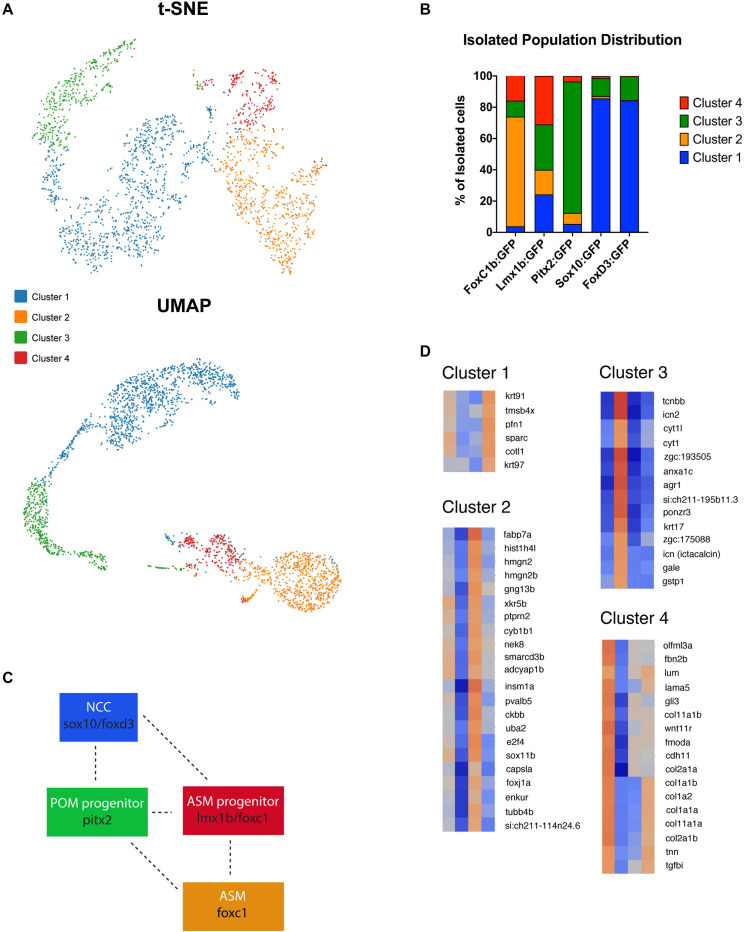
Anterior segment mesenchyme single cell clustering analysis at 48 hpf. **(A)** *K*-means clustering of scRNA sequencing of isolated 48 hpf *foxc1b*:GFP, *lmx1b*:GFP, *sox10:*GFP, *foxd3*:GFP, and *pitx2*:GFP ASM cells in both tSNE and UMAP readouts. Both plots identified four general clusters. **(B)** Distribution of isolated ASM cells within each of the clusters generated by the Cell Ranger3.1 software. **(C)** Model of identified cluster interactions. Interaction and overlap is indicated by dashed lines. **(D)** Heat maps of cluster specific gene expression patterns.

*K*-means clustering (t-SNE) of all five data sets resulted in four distinct clusters (Figure 5A and Supplementary Figure S4). We identified one predominantly NCC-like cluster (Cluster 1) with cells largely originating from the *foxd3* and *sox10* transgenic lines (Figure 5B). 85.6% of *sox10*:GFP and 84.3% of *foxd3*:GFP cells were found in cluster 1. *Sox10*:GFP and *foxd3*:GFP cells were also found in cluster 3 with 11.5 and 15% of their total distribution, respectively. A majority of cells isolated from the *foxc1b:*GFP line were found in Cluster 2, 70.1%, with a small proportion also present in cluster 3, 10.2%, and cluster 4, 15.8% (Figure 5B). *Pitx2:GFP* isolated cells were predominantly found in cluster 3, 83.9%, and cluster 2, 7.1%, with a low proportion found in clusters 1 and 4, 5.3% and 3.6% distribution, respectively. *Lmx1b*:GFP derived cells have the unique classification of being almost equally represented throughout all 4 clusters, 24.3% in cluster 1, 15.7% in cluster 2, 29% in cluster 3 and 31% in cluster 4 (Figure 5B). When comparing spatially, UMAP-based plotting of the clusters indicated a continuous or connected expression profile suggestive of interaction or progression between the clusters (Figure 5A and Supplementary Figure S4). The placement of the clusters within the UMAP graph predicts interaction between *sox10/foxd3* with *pitx2*, *pitx2* and *lmx1b/foxc1b* and finally *lmx1b/foxc1b* with *foxc1b*-derived cells (Figure 5C). Interestingly, cluster 4 appears to represent a transition stage where multiple AS cell fates are possible. Taken together, we observed that none of the clusters are made up solely of cells originating from one individual transgenic line. This observation is supported by our two-color fluorescent *in situ* expression analysis (Figure 2) and is particularly relevant in terms of the relationships amongst POM associated genes, particularly *pitx2* and *foxc1* whose products are actually known to physically interact (Matt et al., 2005; Chawla et al., 2018). Finding four distinct subpopulations of ASM cells further supports our previous findings that suggest ASM are a developmentally heterogenous population during colonization of the AS.

### scRNA Analysis of ASM Uncovers Novel AS Markers

**FIGURE 6:**
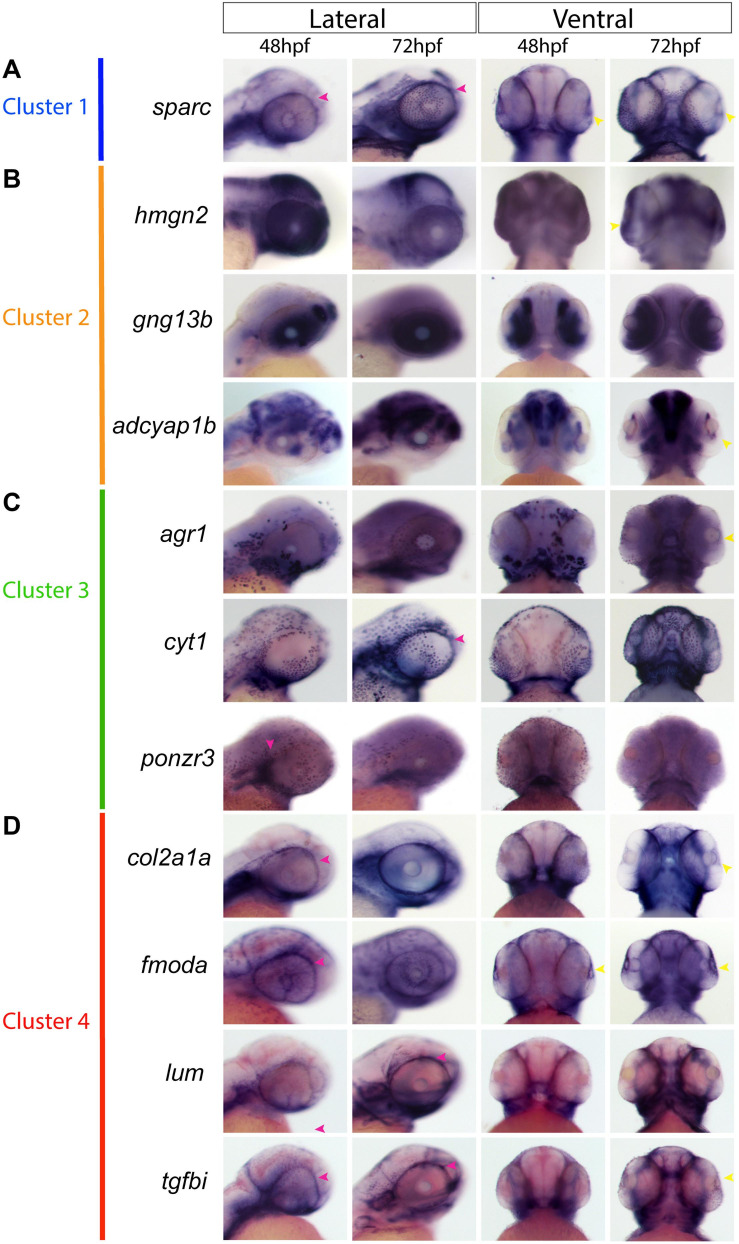
Gene expression of sequencing-derived genes. Whole-mount *in situ* hybridization for novel ASM marker gene mRNA expression patterns was performed during early (48 hpf) and intermediate stages (72 hpf) of AS development and observed in lateral and ventral views. **(A)**, *Sparc* expression representing cluster 1. **(B)** *gng13b*, *hmgn2*, and *adcyap1b* expression representing cluster 2. **(C)** *cyt1*, *agr1*, and *ponzr3* expression representing cluster 3. **(D)** *lum*, *fmoda*, *col2a1a*, and *tgfbi* expression representing cluster 4. Yellow arrowheads indicate AS expression, magenta arrowheads indicate periocular expression.

Based on the clustering analysis we generated heatmaps and gene ontology analysis of the most representative expression patterns for each cluster (Figure 5D and Supplementary Figure S5). Within some of the clusters, genes with possible links to one or more AS structures were identified based on association with ASD-related disorders or potential similarities to POM/NCC-like expression patterns. For example, *cyp1b1*, known to be associated with glaucoma predisposition (Zhao et al., 2015), was identified in cluster 2, while *tgfbi*, isolated in cluster 4, is associated with corneal dystrophy (Poulaki and Colby, 2008). We also noted expression of several collagen (*col2a1a*, *1a1b*, *1a2*, *1a1a*, *2a1b*, *11a1a*, and *11a1b*) and laminin (*lama5*) genes, known to be associated with extracellular matrix properties of AS trabecular meshwork cells (Abu-Hassan et al., 2014), in cluster 4 (Figure 5D). Importantly, our analysis also identified novel candidate genes potentially associated with AS specification and development. We therefore analyzed expression of several potential novel target genes to assess their contribution to AS development at 48 and 72 hpf. Our expression analysis included *sparc* (cluster 1); *gng13b*, *hmgn2* and *adcyap1b* (cluster 2); *cyt1*, *arg1*, *ponzr3*, and *si:ch211-195b11.3* (cluster 3); and *lum*, *fmoda*, *tgfbi*, and *col2a1a* (cluster 4) (Figure 6). Examining expression of cluster 1 associated gene *sparc* revealed that at 48 and 72 hpf *sparc* mRNA was associated with the developing AS, in particular the periphery of the lens and iris regions (Figure 6A). From cluster 2, we examined expression of *hmgn2*, *gng13b*, and *adcyap1b*. We observed strong expression of *hmgn2* throughout the AS by 72 hpf, and strong expression of *adcyap1b* in the lens periphery and future iris region (Figure 6B). Cluster 3 targets included *agr1*, *cyt1*, *ponzr3*, and the currently uncharacterized *si:ch211-195b11.3*. Both *cyt1* and *agr1* appear to label dermal cells in the periocular and AS regions (Figure 6C). *Ponzr3* did not display AS associated expression patterns (Figure 6C), while *si:ch211-195b11.3* does not appear to be related to the AS development (data not shown). Lastly, from cluster 4 we examined *col2a1a*, *fmoda*, *lum*, and *tgfbi* expression. By 72 hpf, we observed strong expression of *fmoda* in the AS as well as some overlapping expression of *tgfbi* and *col2a1a* (Figure 6D). Several genes in our list including *sparc*, *col2a1a*, *lum*, and *tgfbi* also demonstrated periocular expression patterns similar to other known ASM genes at 48 hpf. In particular, *sparc* expression was reminiscent of *sox10*, *lum* expression was reminiscent of *foxc1a* while *tgfbi*, *col2a1a*, and *fmoda* expression were reminiscent of *foxc1b* and *eya2* (Figures 1, 6). In summary, our single cell transcriptomic analysis of ASM cells identified several novel markers associated with early AS formation which may represent uncharacterized regulators of AS development and/or function.

## Conclusion

In the literature, POM has become an umbrella term associated with a host of developmental events including cranial-facial, vascular, retinal as well as anterior segment development. In order to provide some much-needed clarity, our study aimed to characterize specifically the AS associated subset of POM cells, which we term the ASM. As such, our focus was strictly on POM cells that have entered the AS and ultimately contributed to its formation. We used a combination of *in situ* hybridization, real-time imaging of transgenic reporter lines and single cell transcriptomic analysis. Based on our results of ASM distribution, migration dynamics and lastly single cell transcriptional profiles, we conclude that AS colonization employs several distinct, yet developmentally connected, subpopulations of ASM. Overall, our work is the first comprehensive examination of ASM during AS development in zebrafish which we believe will serve as a starting point for future studies of novel regulators of this critical developmental event.

Observing a lack of total co-expression for POM marker genes in ASM cells suggested to us a lack of uniformity within the population. Furthermore, when co-expression of POM markers occurred, the degree and localization within the AS varied between genes examined (Figures 1, 2). *Sox10* and *foxd3*, both NCC markers, appear to be co-expressed in ASM cells distributed over the entire AS at 32 hpf (Figures 2A–C,E,F). Co-expression of *pitx2* and *foxc1* was limited to the periphery of the AS while co-expression of *pitx2* and *eya2* was detected throughout the AS (Figure 2D and Supplementary Figure S2). *Pitx2* displayed a high degree of co-expression with all other POM and NCC markers which likely coincides with its predicted wide-reaching role during AS development. Although we do see some degree of co-localization amongst all POM marker genes, the distinction between each gene’s expression pattern remains evident. Individual ASM cells therefore exhibit a range of POM marker expression patterns which supports our hypothesis that the ASM is heterogenous during AS development. Our findings also speak for the fact that when deciding on a marker for analysis of ASM, not all of the classical POM marker genes may be representative of the entire population and this should be taken into consideration during experimental design.

Utilizing transgenic lines: *foxc1b:*GFP, *foxd3:*GFP, *pitx2:*GFP, *lmx1b.1:*GFP, and *sox10:*GFP we examined the dispersal and distribution of ASM cells during the critical window of POM AS colonization (24–72 hpf). Our detailed imaging studies indicated that the first POM cells arrive on the AS between 22–24 hpf (Figure 3A). GFP+ cells of the *pitx2* subpopulation appear to arrive the earliest of all observed ASM populations, entering the AS as early as 22 hpf, and specifically from the temporal quadrant. When examined as a whole, ASM colonization is, however, primarily dorsal in origin and ASM cells proceed to spread throughout the AS as development proceeds. This pattern was observed for *foxc1b*:GFP, *foxd3*:GFP, *sox10*:GFP, and *lmx1b*:GFP ASM cells. In addition to several differing entry points, we also observed varying targeting behavior of ASM cells. By 48 hpf homogenous AS distribution was observed for *foxc1b*:GFP, *foxd3*:GFP, and *sox10*:GFP cells. Conversely, *lmx1b.1*:GFP and *pitx2*:GFP cells remained strictly restricted to the nasal and temporal quadrants of the AS, respectively. This is a clear indication that heterogeneity during AS colonization is also a potential mechanism for specific targeting within the AS. Lastly, we observed that migratory ASM are not actively undergoing cellular division. The rapid increase in ASM cell number between 24–48 hpf within some subpopulations, in particular *foxc1b:*GFP and *foxd3:*GFP, may therefore be primarily attributed to the continual arrival of migratory cells to the AS. This notion is supported by the fact that cranial NCCs, the main source of POM/ASM, originate dorsal to the AS and that we observe *foxd3*:GFP and *foxc1b*:GFP cells entering the AS predominantly within the dorsal quadrant. Alternatively, it remains possible that GFP+ cells represent a non-proliferative pool of ASM. Analyzing co-expression of ASM markers and cell cycle transcripts will be necessary to test this alternative. When combining all of our ASM distribution data, we propose a progressive colonization model outlined in Figure 3C.

The ability to migrate long distances and respond to specific cues is a crucial and well documented behavior of NCCs. Cranial NCCs migrate without designated leader or follower identities, instead maintaining a large homogenous population wherein each member exhibits the same migratory capabilities as its neighbors (Clay and Halloran, 2010; Kulesa et al., 2010; Richardson et al., 2016). 4D live imaging of *foxc1b*:GFP, *foxd3*:GFP, *sox10*:GFP, *pitx2*:GFP, and *lmx1b.1*:GFP transgenic embryos aged 24–48 hpf showed a uniform migration behavior pattern in ASM subpopulations indicative of cranial neural crest-like migration (Supplementary Movies S1–S5). Tracking analyses indicated cells had stochastic and independent migratory capabilities, frequently pausing during migration, altering directionality, and extending filipodia to communicate with one another (Supplementary Movies S6–S10). Quantification of total distance and average velocity indicated that *foxc1b:*GFP ASM cells traveled the farthest distance and with the highest velocity (Figures 4C,D). Conversely, *pitx2:*GFP ASM cells traveled the shortest distances and with the slowest velocities, but also with the least amount of stochastic movement (Figures 4B–D). Interestingly, since *pitx2*:GFP+ cells are the first POM cells to colonize the AS, we hypothesize that they may serve as sentinels to mark the AS for later arriving POM. Their need for timely arrival at the AS may explain their unique, highly targeted, migratory behavior. Displacement, also referred to as directed migration, was measured as a way to characterize the purposeful migration of ASM cells. We wanted to quantify whether certain ASM cells migrated with more directionality than their counterparts. Interestingly, our data indicates that cells in the *sox10*:GFP and *foxd3*:GFP subpopulation, associated with NCC identity (Stewart et al., 2006; Drerup et al., 2009; Wang et al., 2011), engage the most in directed migration (Figure 4E). These likely pluripotent, NCC-like cells may be directly targeting to specific regions of the AS in order to ensure equal distribution. Cells likely associated with a more traditional POM identity, *foxc1b*:GFP and *pitx2*:GFP (Liu and Semina, 2012; Reis et al., 2012), appear to have more stochastic migration paths (Figure 4E). A more stochastic migration pattern may be indicative of ASM cells further along the differentiation spectrum and no longer needing to target to specific AS regions. Similar to our observations of ASM expression patterns and distribution (**Figures 1**–3), migratory behavior is also clearly variable amongst ASM cells which further supports the notion of a heterogenous population.

Lastly, we investigated the transcriptomic differences amongst the ASM. Utilizing the 10× Genomics process of scRNA sequencing, we isolated 48 hpf eyes from transgenic embryos (*foxc1b*:GFP, *foxd3*:GFP, *lmx1b*:GFP, *sox10*:GFP, and *pitx2*:GFP) and used FACS to isolate our GFP+ ASM cells. This approach aimed to ensure only AS associated cells were included in our analysis. ASM specific single cell cDNA libraries were generated, sequenced, and based on transcriptomic profiles, grouped into four distinct clusters (Figures 5A,B). We classified these clusters into a NCC-like cluster (*sox10/foxd3*), POM progenitor cluster (*pitx2*), a ASM progenitor cluster (*lmx1b/foxc1b*) and finally a specified ASM cluster (*foxc1b*) (Figures 5B,C). UMAP analysis of the data indicates a connection between clusters suggesting that ASM cells may be found along a heterogenous, but connected, spectrum during AS colonization. This connection is also apparent when examining the cluster associated percentage distribution of our isolated cells. The four clusters all appear to be connected along a plausible trajectory of AS development starting with the NCC-like cluster (cluster 1), then the POM progenitor cluster (cluster 3) followed by the ASM progenitor cluster (cluster 4) and finally an ASM-like cluster (cluster 2). We predict that early AS is therefore likely colonized by a heterogenous population of ASM that may represent different stages of differentiation along the path to an AS cell fate. This combination could ensure that while some ASM enter already poised to begin differentiation and assembly of functional AS structures, such as the *pitx2*, *foxc1b*, or *lmx1b* driven subpopulations, others that are likely more pluripotent, *foxd3/sox10* driven, serve to ensure that proper numbers of ASM arrive at all points along the AS and then further differentiate in response to local signaling cues. It is therefore not surprising that distributions, migratory patterns and transcriptomic profiles of *sox10:*GFP and *foxd3:*GFP cells are highly similar when compared to *foxc1b:*GFP, *lmx1b.1:*GFP, or *pitx2:*GFP. Finally, while transcriptomic differences observed in our data support the notion of a heterogenous ASM population, they do not infer lineage marking. Future fate mapping experiments will need to be performed to determine whether specific transcriptomic differences directly determine cell fate or simply represent transitional states along the ASM differentiation pathway.

Expression analysis of cluster specific targets identified by our scRNA assay revealed a number of genes of particular interest including *sparc*, *hmgn2*, *adcyap1b*, *agr1*, *col2a1a tgfbi*, and *fmoda* (Figure 6). Several of these genes are associated with regulation and function of the extracellular matrix including *sparc*, *col2a1a*, and *fmoda*, indicating a possible relationship with the drainage systems of the iridocorneal angle (Abu-Hassan et al., 2014). While the drainage networks are the last to fully differentiate in the AS, their importance to the function of the eye cannot be understated. *Tgfbi* is associated with head mesenchyme and neural crest and is also predicted to be localized to the extracellular matrix (Chakravarthi et al., 2005; Kannabiran and Klintworth, 2006). Interestingly, the t*gfbi* gene is associated with a subset of familial corneal dystrophies (Nielsen et al., 2020). *Hmgn2* is a non-histone component of nucleosomal DNA and is associated with transcriptionally active chromatin, especially in cells that remain undifferentiated, and is involved in DNA replication, transcription, and repair (Lucey et al., 2008; Furusawa and Cherukuri, 2010). Although not yet associated with AS development or ASD pathology, *hmgn2* is abundantly expressed throughout the ocular structures of the AS and retina in mice, especially in the lens fibers and epithelium layer of the cornea (Lucey et al., 2008). *Adcyap1b* is a neuropeptide also known as PACAP associated with brain and camera-type eye development, meaning that it plays a role in the development of the lens and retina of the eye (Wu et al., 2006; Denes et al., 2019). PACAP also appears to have retinoprotective attributes (Shioda et al., 2016). The mechanisms of *adcyap1b* function during AS development is unclear at this time. Finally, *agr1* is associated with the negative regulation of cell death, regenerative abilities of fish and frogs and is strongly associated with liver function (Ivanova et al., 2015, 2018). While it’s function within the eye remains unclear at this time, *agr1* does show expression in the corneal region (Figure 6) (Ivanova et al., 2015). Future studies will focus on examining the functional roles of the aforementioned AS associated target genes as well as others from our transcriptomic screen (Figure 5D). We will also seek to determine whether the identified clusters also represent lineages of specific AS structures, such as the cornea, iridocorneal angle (annular element in zebrafish) or iris.

Heterogeneity within the early colonizing POM and subsequent ASM poses an important question: what is the functional interplay between such subpopulations? Previous studies of AS development in several models suggest that there are in fact “master regulators” of this process, in particular *foxc1* and *pitx2*. Loss of function of either has significant consequences on AS formation and function. However, the status of other POM/ASM markers, or subpopulations, in these circumstances has not been thoroughly examined. It therefore remains unknown whether all ASM subpopulations are affected and to what degree. We know even less about the consequences of other POM/ASM regulators, such as *eya2*, *foxd3*, or *lmx1b*, in this context. Future work will need to concentrate on carefully teasing out how individual regulators of POM influence the entire process of AS colonization, rather than simply observing for physiological consequences in juvenile or adult AS tissues.

In conclusion, our findings, based on distribution, migration and transcriptomic profiles, indicate that POM cells targeted to the anterior segment, which we have termed the Anterior Segment Mesenchyme (ASM), are not homogenous. Rather, the ASM simultaneously comprises of several subpopulations likely divided along the ASM differentiation pathway. Our findings open a wide range of possible new investigative paths in the area of AS development and ASD disorders. Future examination of the interplay between these subpopulations will be prudent to further our understanding of eye development and ultimately predisposition to ASD associated blinding disorders.

## Materials and Methods

### Zebrafish Maintenance

Zebrafish lines were bred and maintained in accordance with IACUC regulations (IACUC protocol 2015-1380) at the University of Kentucky. AB strain was used as wildtype. Transgenic lines used were: Tg[*foxc1b:GFP]* (Dr. Bryan Link), Tg[*foxd3:GFP]* (Dr. Lister), Tg[*pitx2C4:GFP]* (Dr. Elena Semina), and Tg[*lmx1b.1:GFP]* (Dr. Brian Link), Tg[*sox10:RFP]* (Dr. Lister). All embryos were raised for the first 24 h post fertilization in embryo media (E3) at 28°C. After 24 h, E3 media was replaced with embryo media containing 1-phenyl 2-thiourea (PTU) every 24 h to maintain embryo transparency.

### Whole-Mount *in situ* Hybridization (WISH)

Whole-mount *in situ* hybridizations were performed using a minimum of 20–25 embryos for each time point (12, 18, 24, 32, 48, and 72). DIG and FITC labeled RNA probes were created using PCR incorporating T7 promoters in the primers and transcribed with T7 polymerase (Roche). Forward and Reverse primer sequences are listed in Supplementary Table S1. WISH protocol was performed as previously described (Holly et al., 2014). Dorsal, lateral, and ventral images of embryos were captured using a Nikon Digital Sight DS-U3 camera and Elements software. Images were adjusted for brightness using Adobe Photoshop and assembled into figures using Adobe Illustrator.

### Immunohistochemistry (IHC) for Distribution and Proliferation Analysis

Approximately 30 embryos were imaged for each transgenic line at each of the given time points (24, 26, 28, 30, 48, 54, and 72 hpf). Embryos were fixed overnight at 4°C using 4% PFA. PFA was washed out with PBST 4 times for 5 min each. Embryos were permeabilized with Proteinase K (10 μg/ml) at the following times (24 hpf = 5 min; 26 hpf = 6 min; 28 hpf = 7 min; 30 hpf = 9 min; 48 hpf = 20 min; 54 hpf = 25 min; 72 hpf = 40 min), washed with PBST and then blocked with 5% goat serum (1 g/100 ml), 1% BSA in a solution of 1x PBST for at least 2 h at room temperature. Primary antibody (Rockland rabbit anti-GFP) was diluted at 1/200 in blocking buffer and incubated overnight at 4°C on rotation. The following day, the primary antibody solution was washed out with PBST 5 times for 15 min each. Secondary antibody (Alexa Fluor 488 anti rabbit, 1/1000) and DAPI (1/2500) were diluted in blocking buffer and incubated for 1 h on rotation in the dark at room temperature. Embryos were washed 2× for 15 min with PBST in the dark.

After staining, embryos were embedded in a 1.2% Low-gelling agarose in a 1-inch glass bottom cell culture dish (Fluorodish, World Precision Instruments) and visualized using a Nikon C2+ confocal microscope with a 20× (0.95 NA) oil immersion objective. The anterior segment of the eye was imaged in 3D in the lateral position as a 100 μm z-stack using 3.50 μm steps. All images were captured using Nikon Elements software, adjusted for contrast and brightness using Adobe Photoshop and assembled into figures using Adobe Illustrator. Images generated from IHC analysis were rendered in 3D using Nikon Elements Viewer software. Eyes were divided into 4 quadrants: dorsal nasal, dorsal temporal, ventral nasal, and ventral temporal. Nasal and temporal regions were divided by a vertical straight line through the center of the lens, while dorsal and ventral were divided by a horizontal straight line through the center of the lens. For distribution analysis, GFP+ cells were manually counted based on their position within one of the four quadrants of a 3D constructed anterior segment. For each timepoint 25+ embryos from three independent trials were imaged for quantification.

### Two-Color Fluorescent WISH

RNA probes were generated using the MEGAscript T7 transcription Kit (Ambion) in combination with RNA labeling mixes for both DIG and FITC (Roche). Double *in situ* hybridization was performed according to the protocol by Lauter et al. (2011). This included exposing embryos to acidified methanol and adding Dextran Sulfate into the hybridization reaction. Staining was done by combining Fast Blue and Fast Red dyes (Sigma) (50 μg/ml). After successful *in situ* double staining, embryos were additionally stained using DAPI and imaged using a NIKON C2+ confocal microscope. Images were adjusted for brightness and contrast using Adobe Photoshop and assembled into figures using Adobe Illustrator. 15–20 embryos were analyzed for each probe combination.

### Time-Lapse Confocal *in vivo* Imaging

Embryos from each of the previously mentioned transgenic lines were collected and raised in E3 media at 28°C. Fluorescent embryos were placed in E3 PTU media including 3-amino benzoic acid ethyl ester (Tricaine) to prevent pigmentation and to anesthetize them, respectively. They were then dechorionated and embedded laterally in 1% low-gelling agarose in a 35 mm glass bottom cell culture dish (Fluordish, World Precision instruments). Real-time imaging was conducted at 28°C using a Nikon C2+ confocal microscope and a 20× (0.95 NA) oil immersion objective. 3D *z*-stacks over a 75 μm thickness with a slice size of 3.5 μm were collected to encompass the entire developing anterior segment. *Z*-stack images were taken at 10 min intervals over a 24 h period (embryos imaged: *n* = 12 *foxc1b:GFP*, *n* = 9 *foxd3:GFP*, *n* = 13 *pitx2:GFP*, *n* = 10 *lmx1b.1:GFP*, *n* = 7 *sox10:RFP*). Data were collected and rendered using Nikon Elements software. Images were adjusted for brightness using Adobe Photoshop and assembled into figures using Adobe Illustrator.

### Cell Migration Tracking and Displacement Analysis

Completed 4D live imaging files were uploaded into FIJI software for analysis. Approximately 25 cells were manually tracked per video file using manual tracking tools. Tracked cells were measured for total distance traveled (μm), average velocity (μm/min), and total displacement (pixels). Tracked cells were randomly selected from all four eye quadrants to ensure all eye regions were represented, as well as all time frames. After tracking, data were exported to Microsoft Excel for statistical analysis. Displacement was measured using the line measurement tool in FIJI software. Previously tracked lines were identified as “completed” tracks in one of two ways: (1) at the end of the migration video or (2) the track was seen in the last frames of video before the specific track disappeared. Once the completed track was identified, the line measurement tool was used to measure the straight-line distance (in pixels) from the first point of the track to the last point of the track. Stats were analyzed in Microsoft Excel and Graphpad Prism8.

### Single Cell Transcriptomic Analysis

Embryos from each of the POM subpopulation transgenic lines were dechorionated and incubated in E3 media at 28°C until 48 hpf. At this time, embryos were anesthetized using 3-amino benzoic acid ethyl ester (Tricaine) and their eyes dissected and collected on ice. Eyes were incubated for 2 min in 0.25% Trypsin + EDTA at 37°C. After incubation, a 20G needle and syringe were used to dissociate the tissue before the tube was placed back at 37°C for 2 min. This process was repeated four times. After incubation, the dissociated cells were strained using a 40 μm filter (VWR) and spun down for 10 min at 3,500 rpm at 4°C. The supernatant was removed and the pellet resuspended in 1x PBS + 2 mM EDTA and goat serum. Cells were sorted for GFP+ identity at the University of Kentucky Flow Cytometry and Immune Monitoring Core at the Markey Cancer Center. After sorting, cells were spun down and resuspended using PBS and goat serum. Approximately 1,000 cells from each transgenic line were then loaded onto the Chromium 10× V3 chip (10× genomics) and processed in the University of Kentucky Department of Biology Imaging Core to generate single cell barcoded cDNA. Sequencing was performed using NovaSeq SP, 2 × 150 bp paired ends to achieve 100,000 reads per cell at University of Illinois at Urbana-Champaign Roy J. Carver Biotechnology Center. Sequencing results were processed and subsequently aggregated (incorporating mapped normalization), using the Cell Ranger3.1 pipeline and results analyzed using Loupe Cell Browser 3.1.1 software (10× genomics).

### Statistics

One-way ANOVA analysis (multiple point analysis) and unpaired *t*-tests (individual comparison analysis) were performed using Microsoft Excel and GraphPad Prism8 software. All graphs are shown with their respective means and standard deviations. Values were considered significant by the conventional standard: *P-*value of 0.05 or less.

## Data Availability Statement

The raw data supporting the conclusions of this article will be made available by the authors, without undue reservation, to any qualified researcher.

## Ethics Statement

The use of zebrafish in this study was approved by the University of Kentucky IACUC committee, Institutional PHS Assurance #D16-00217 (A3336-01) with a protocol number: 2015-1370. All experimental protocols were approved by the University of Kentucky Institutional Biosafety Committee, registration number B18-3186-M.

## Author Contributions

JF and KV wrote the manuscript. KV, OV, MW, and NM performed the experiments and analysis. JF oversaw the project secured funding. JF and KV contributed to conception and design of the study. All authors contributed to manuscript revision, read and approved the submitted version.

## Conflict of Interest

The authors declare that the research was conducted in the absence of any commercial or financial relationships that could be construed as a potential conflict of interest.

## Supplementary Material

FIGURE S1 Schematic representation of AS colonization in a zebrafish embryo. Upon closure of the neural tube (NT) neural crest cells (NCC) delaminate and begin to migrate throughout the embryo (12–18 hpf), including the cranial NCC within the developing head (pictured as black outline; OV, optic vesicle). Upon retinal morphogenesis and lens (L) induction (18–28 hpf), NCC begin to surround the retina (R) in anticipation of AS colonization and become periocular mesenchyme (POM). POM targeted to the AS subsequently migrate onto the surface of the retina and occupy the future AS (28–48 hpf).

FIGURE S2 Anterior segment ASM heterogeneity for *eya2* expression. **(A–D)** Two-color fluorescent WISH (FWISH) performed for all possible combinations of *eya2* vs. *foxc1a*, *foxd3*, *pitx2*, and *sox10* at 32 and 48 hpf. DAPI is in blue. White arrows within inset panels (dashed squares) display instances of individual **(b)** and co-expression **(a)**. Scale bar = 50 μm.

FIGURE S3 PH3 proliferation staining assay. 3D rendering of confocal stacks of the AS at 32 and 48 hpf. GFP+ cells (green) are the result of transgenic lines, pH3+ cells (red) indicate active cell division, and DAPI (blue) stains the nucleus. **(A)** Few pH3+ cells can be seen on the surface of the AS at 32 hpf, indicating little to no cell division is taking place. No co-expression was seen between the GFP+ cells and pH3+ cells in any of the POM subpopulations. **(B)** Similarly, the AS at 48 hpf also lacked pH3+ cells and had little to no co-expression with the various POM subpopulations.

FIGURE S4 cDNA library-based t-SNE and UMAP clusters. *K*-means clustering of scRNA sequencing of isolated 48 hpf *foxc1b:*GFP (pink), *lmx1b.1:*GFP (green), *sox10:*GFP (blue), *foxd3:*GFP (mustard) and *pitx2*:GFP (teal) ASM cells in both tSNE and UMAP readouts.

FIGURE S5 PANTHER Gene Ontology for 48 hpf ASM clusters. Protein function Gene Ontology was applied to gene lists from clusters 1–4. Pie graphs display the distribution of each GO term applied.

TABLE S1 *WISH* Primer Sequences. mRNA forward and reverse primer sequences for all POM and NCC-related genes.

TABLE S2 scRNA sequencing aggregation gene list. Distribution of all gene expression analyzed during aggregation analysis.

MOVIE S1 *Foxc1b:GFP* 4D imaging (24–28 hpf).

MOVIE S2 *Foxd3:GFP* 4D imaging (24–48 hpf).

MOVIE S3 *Pitx2:GFP* 4D imaging (22–46 hpf).

MOVIE S4 *Lmx1b.1:GFP* 4D imaging (24–48 hpf).

MOVIE S5 *Sox10:RFP* 4D imaging (23–47 hpf).

MOVIE S6 *Foxc1b:GFP* Tracking analysis.

MOVIE S7 *Foxd3:GFP* Tracking analysis.

MOVIE S8 *Pitx2:GFP* Tracking analysis.

MOVIE S9 *Lmx1b.1* Tracking analysis.

MOVIE S10 *Sox10:RFP* Tracking analysis.
