# Supplementary figures and images for: Spatiotemporal Characterization of Anterior Segment Mesenchyme Heterogeneity During Zebrafish Ocular Anterior Segment Development
Source: Front Cell Dev Biol. 2020 May 27;8:379. doi: 10.3389/fcell.2020.00379 (PMC7266958; doi:10.3389/fcell.2020.00379)

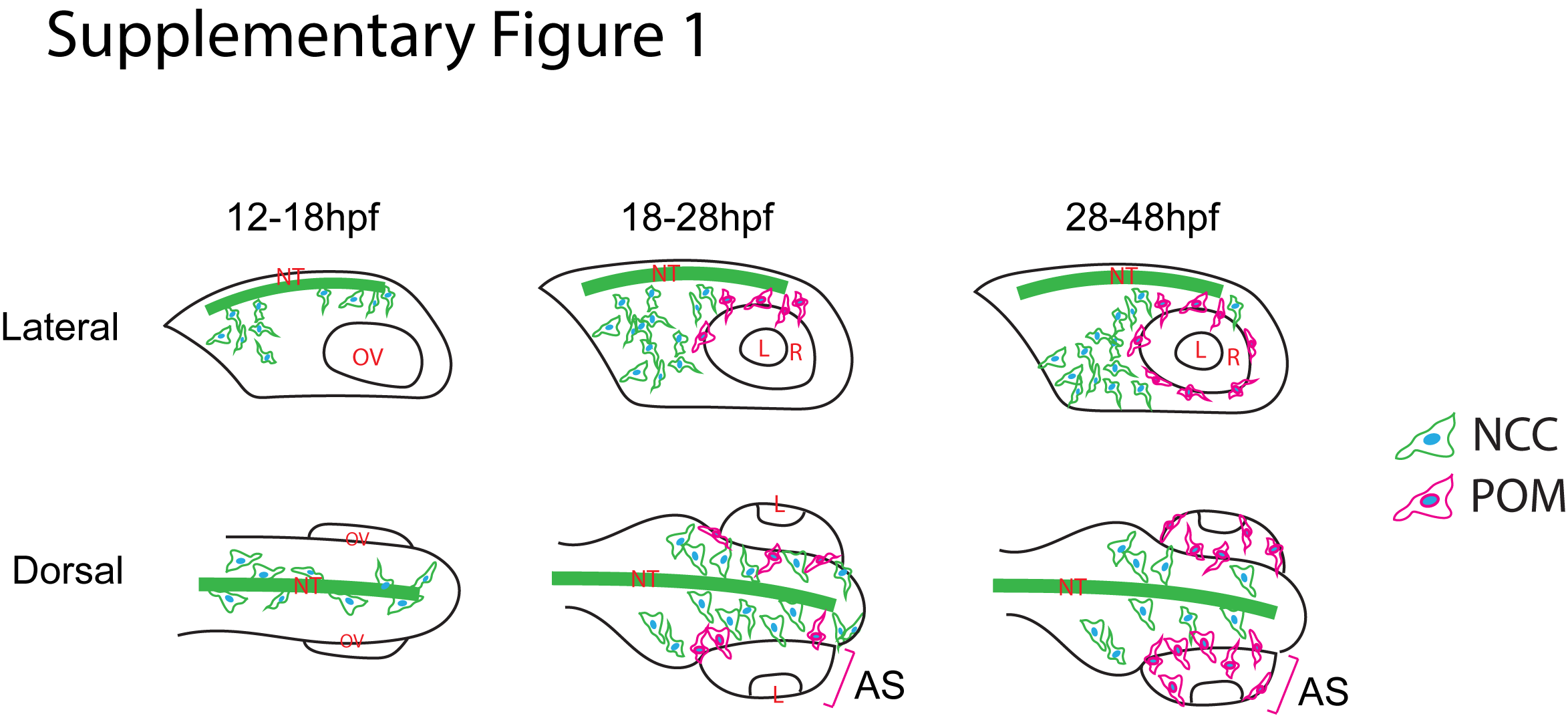

Supplement: FIGURE S1 — Schematic representation of AS colonization in a zebrafish embryo. Upon closure of the neural tube (NT) neural crest cells (NCC) delaminate and begin to migrate throughout the embryo (12–18 hpf), including the cranial NCC within the developing head (pictured as black outline; OV, optic vesicle). Upon retinal morphogenesis and lens (L) induction (18–28 hpf), NCC begin to surround the retina (R) in anticipation of AS colonization and become periocular mesenchyme (POM). POM targeted to the AS subsequently migrate onto the surface of the retina and occupy the future AS (28–48 hpf). [file Image_1.tif]

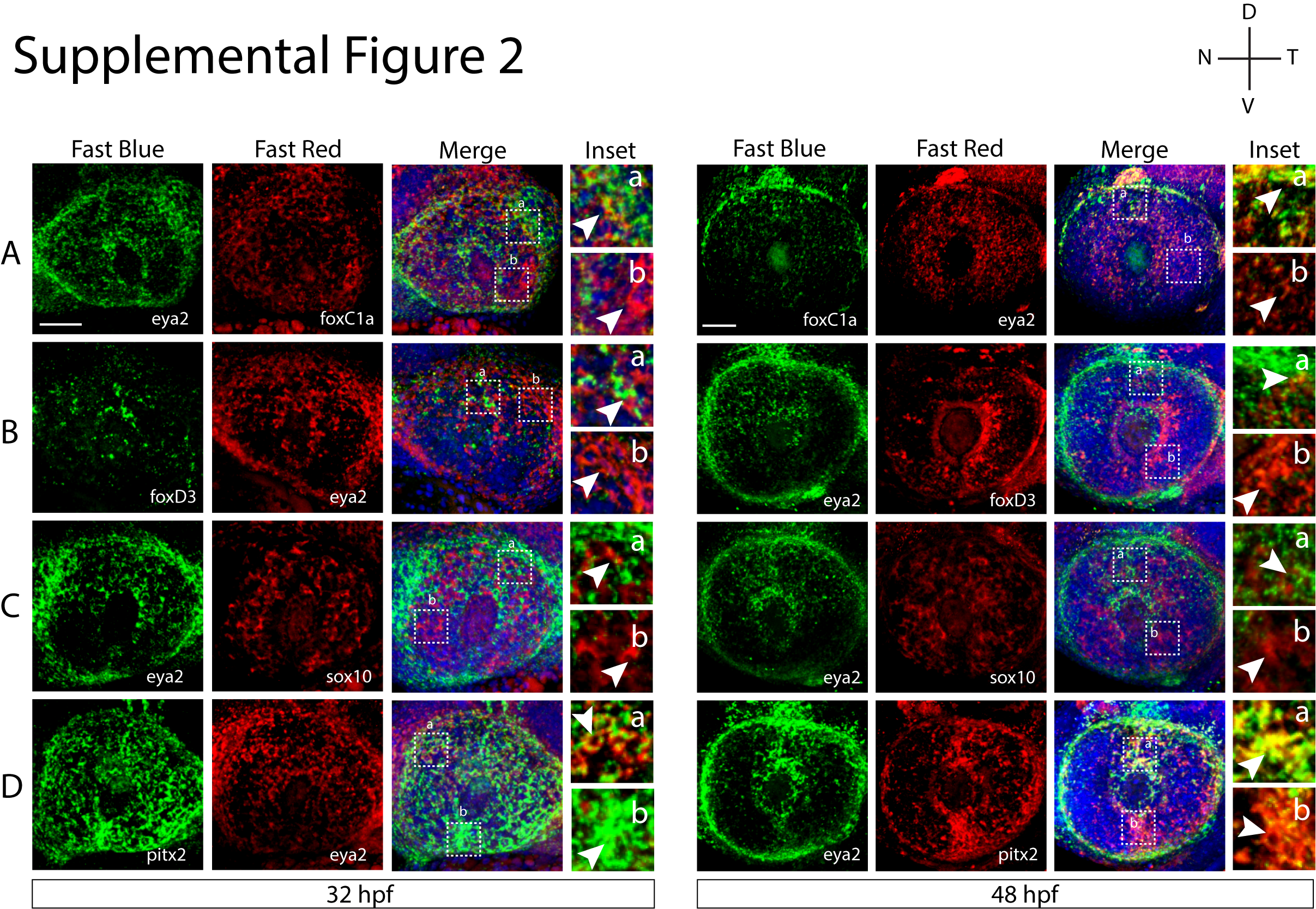

Supplement: FIGURE S2 — Anterior segment ASM heterogeneity for eya2 expression. (A–D) Two-color fluorescent WISH (FWISH) performed for all possible combinations of eya2 vs. foxc1a, foxd3, pitx2, and sox10 at 32 and 48 hpf. DAPI is in blue. White arrows within inset panels (dashed squares) display instances of individual (b) and co-expression (a). Scale bar = 50 μm. [file Image_2.tif]

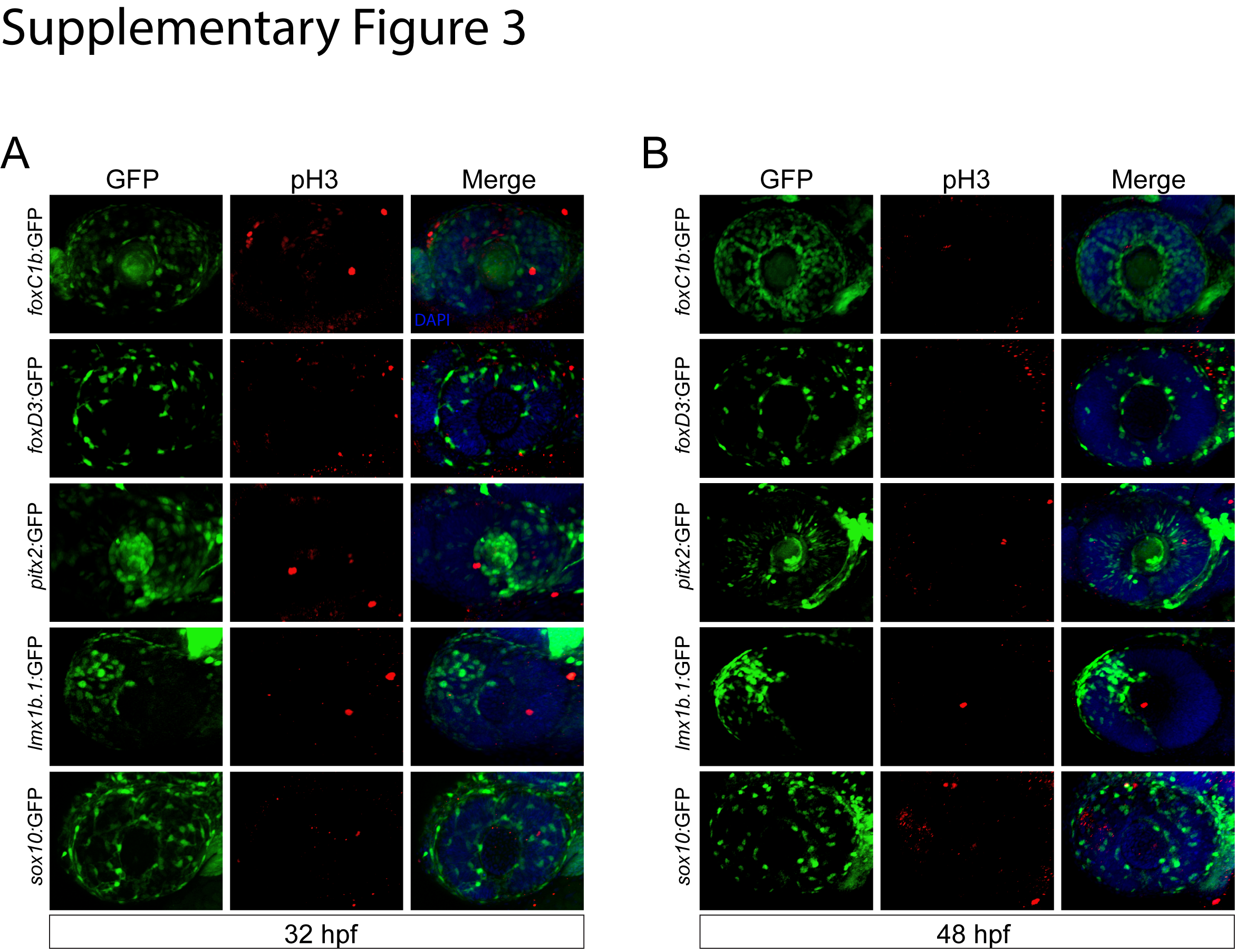

Supplement: FIGURE S3 — PH3 proliferation staining assay. 3D rendering of confocal stacks of the AS at 32 and 48 hpf. GFP+ cells (green) are the result of transgenic lines, pH3+ cells (red) indicate active cell division, and DAPI (blue) stains the nucleus. (A) Few pH3+ cells can be seen on the surface of the AS at 32 hpf, indicating little to no cell division is taking place. No co-expression was seen between the GFP+ cells and pH3+ cells in any of the POM subpopulations. (B) Similarly, the AS at 48 hpf also lacked pH3+ cells and had little to no co-expression with the various POM subpopulations. [file Image_3.tif]

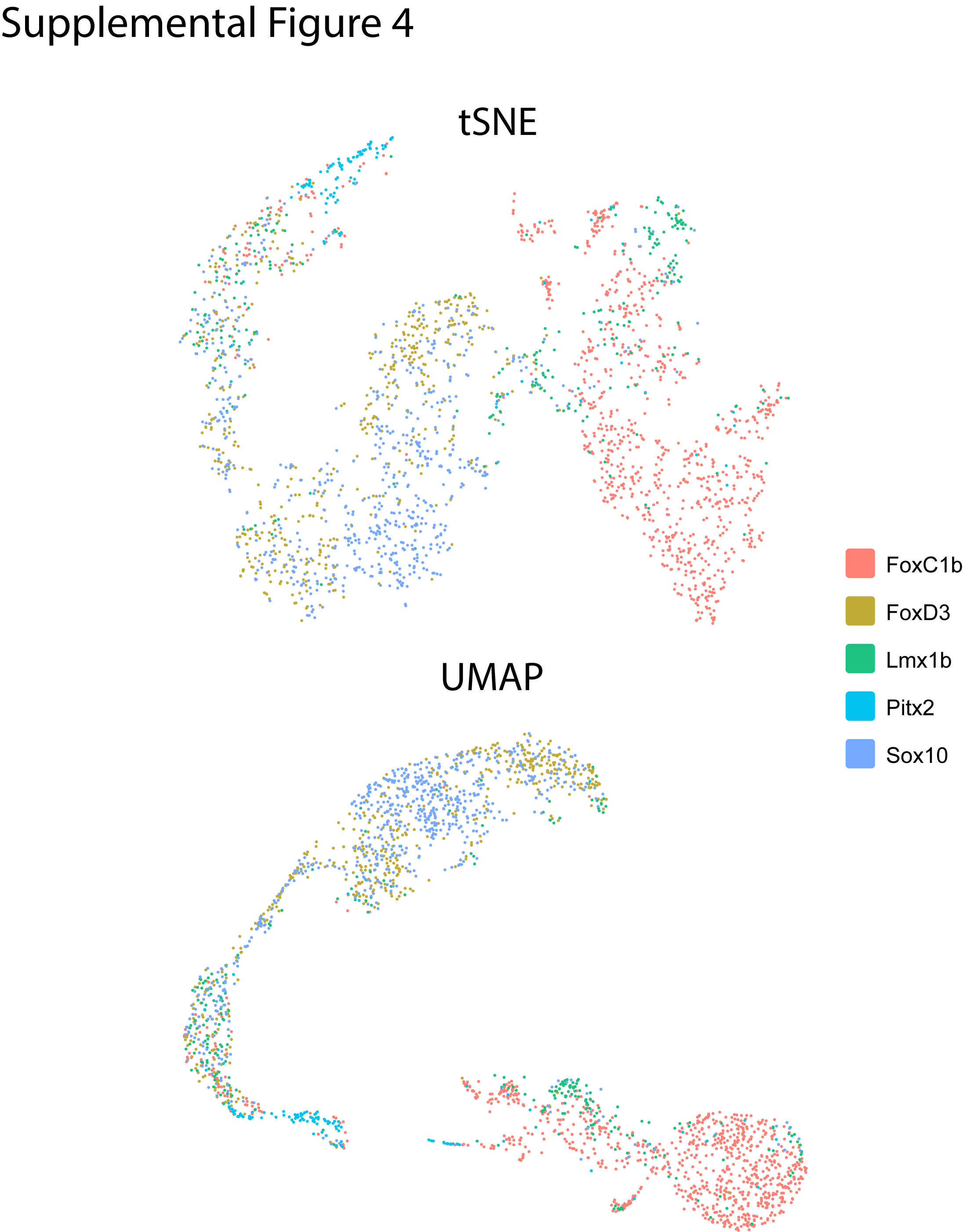

Supplement: FIGURE S4 — cDNA library-based t-SNE and UMAP clusters. K-means clustering of scRNA sequencing of isolated 48 hpf foxc1b:GFP (pink), lmx1b.1:GFP (green), sox10:GFP (blue), foxd3:GFP (mustard) and pitx2:GFP (teal) ASM cells in both tSNE and UMAP readouts. [file Image_4.tif]

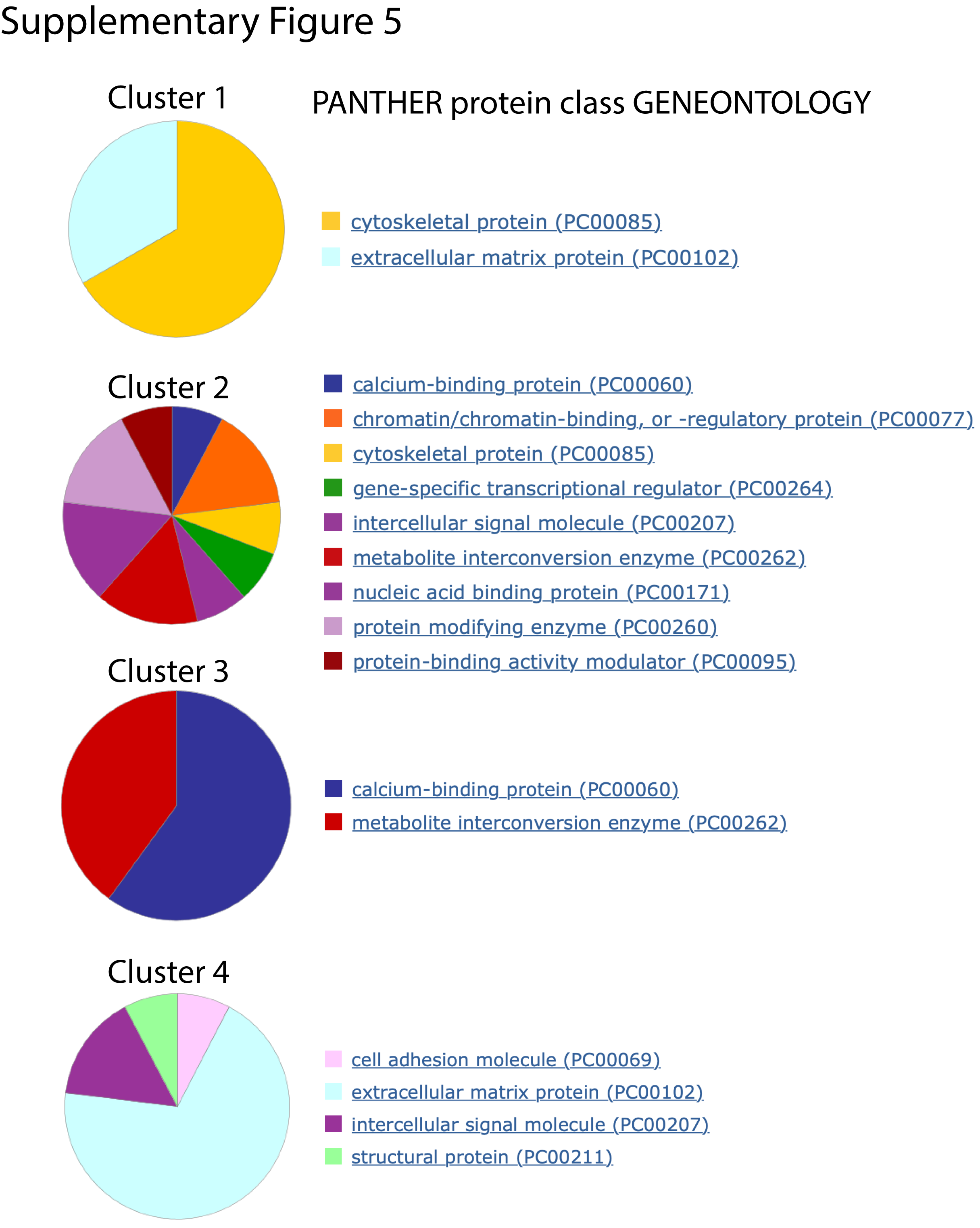

Supplement: FIGURE S5 — PANTHER Gene Ontology for 48 hpf ASM clusters. Protein function Gene Ontology was applied to gene lists from clusters 1–4. Pie graphs display the distribution of each GO term applied. [file Image_5.tif]
